# Genetic and Antigenic Characterization of Enterovirus 71 in Ho Chi Minh City, Vietnam, 2011

**DOI:** 10.1371/journal.pone.0069895

**Published:** 2013-07-29

**Authors:** Le Phan Kim Thoa, Pai-Shan Chiang, Truong Huu Khanh, Shu-Ting Luo, Tran Ngoc Hanh Dan, Ya-Fang Wang, Tang Chi Thuong, Wan-Yu Chung, Nguyen Thanh Hung, Jen-Ren Wang, Le Nguyen Thanh Nhan, Le Quoc Thinh, Ih-Jen Su, Than Duc Dung, Min-Shi Lee

**Affiliations:** 1 Children’s Hospital No. 1, Ho Chi Minh City, Vietnam; 2 National Institute of Infectious Diseases and Vaccinology, National Health Research Institutes, Zhunan, Taiwan; 3 College of Medicine, National Cheng Kung University, Tainan, Taiwan; Johns Hopkins School of Public Health, United States of America

## Abstract

Enterovirus 71 (EV71) frequently causes fatal infections in young children in Asia. In 2011, EV71 epidemics occurred in southern Vietnam. We conducted genetic and antigenic analysis of the EV71 isolates and found that 94% of them were genotype C4a related to two lineages circulating in China and 6% were genotype C5 which have circulated in Vietnam since 2003. Antigenic variants were not detected. EV71 vaccines are being developed. Longitudinal enterovirus surveillance data are critical to formulate vaccination policy in Vietnam.

## Introduction

Enterovirus 71 (EV71) is a member of genus Enterovirus within the family Picornaviridae. Although EV71 was first described in 1969, a retrospective analysis shows that this virus circulated in the Netherlands as early as 1963 [1]. Recent molecular evolution studies predicted that EV71 could emerge in human population around 1941 and seems to evolve more quickly through gene recombination in the past 15 years [2,3]. Based on VP1 genotyping, EV71 could be classified into 3 genogroups (A, B and C) including 11 genotypes (A, B1 ~ B5, C1 ~ C5). The C4 genotype could be further classified into C4a and C4b [3]. Recently, three new genogroups (genogroup D, E, and F) were identified in India [4]. Since 1997, EV71 has repeatedly caused life-threatening outbreaks of hand-foot-mouth disease (HFMD) with neurological complications in Asian children. The neurological manifestations progress very quickly and range from aseptic meningitis to acute flaccid paralysis and brainstem encephalitis, which frequently cause societal panics [3].

In Vietnam, EV71 was first identified in 2003 and a large EV71 epidemic was documented in southern Vietnam in 2005, which reported 173 EV71 cases including 51 with neurological complications and 3 fatal cases [5]. Based on phylogenetic analysis of complete VP4 and partial VP1 genes, 94% (162/173) of the 2005 isolates were attributable to a novel genotype C5, 5% (9/173) were genotype C4, and 1% (2/173) were genotype C1. Twenty-three strains (16 C5, 5 C4 and 2 C1) were further confirmed with analysis of complete VP1 gene. However, full genome of C5 viruses isolated in Vietnam is not available in public domain, which is critical to elucidate its molecular evolution. From 2006 to 2010, only sporadic EV71 cases were detected in southern Vietnam [6]. In 2011, EV71 epidemics occurred again in southern Vietnam. Clinical characterization of the 2011 epidemic has recently been described [7]. In this study, we conducted genetic and antigenic analysis of the EV71 strains isolated in a children hospital in Ho Chi Minh City, Vietnam, 2011.

## Methods

### Study populations

Children’s Hospital No. 1 (CH1), Ho Chi Minh (HCM) City, is a major pediatric hospital serving children in HCM City and southern Vietnam. In CH1-HCM, clinical specimens from hospitalized children with HFMD were collected for virus isolation. Institutional review board approvals were obtained from CH1-HCM following the Helsinki Declaration; and written informed consent was obtained from guardians of participating children.

### Clinical and laboratory definitions

Clinical stages of enterovirus cases in Vietnam are defined as follow [7]: Stage I is uncomplicated disease such as herpangina or HFMD; IIA is myoclonus reported by the caregiver; IIB is myoclonus observed by a physician; III is autonomic dysfunction with fever that is not responsive to antipyretics and with hypertension and persistent tachycardia; IV is cardiopulmonary compromise with pulmonary edema or hemorrhage. Laboratory evidence of EV71 infection was defined as the isolation of EV71 from a throat swab, a rectal swab, or a stool sample.

### Virologic analysis

In CH1-HCM, clinical samples (throat swabs, or rectal swabs) were routinely collected for virus isolation from hospitalized pediatric patients with suspected enterovirus infections (HFMD or encephalitis). Samples were inoculated into rhabdomyosarcoma cells. When enteroviral cytopathic effect involved more than 50% of the cell monolayer, cells were scraped and subjected to indirect fluorescent antibody staining with pan-enterovirus and EV71-specific monoclonal antibodies (3360, 3324, Millipore, USA). VP1 genes of isolated EV71 viruses were sequenced and genotyped by phylogenetic analysis using the Neighbor-joining method in MEGA 4 software as described previously [8]. Full genomes of selected EV71 viruses were sequenced for phylogenetic analysis and detection of gene recombination [9–11]. Backgrounds of reference virus sequences used in the phylogenetic analysis were listed in the Table S1. Primers for amplifying and sequencing VP1 genes and complete genome were listed in Table S2.

### Serologic assays

Post-infection sera were collected from Taiwanese children infected with EV71 for measuring neutralizing antibody titers [12,13] (Table 1). Laboratory methods for measuring EV71 serum neutralizing antibody titers followed standard protocols [14]. Twofold serially diluted sera (1:8 -1:512) and virus working solution containing 100 TCID_50_ of EV71 strain, were mixed on 96-well microplates and incubated with rhabdomyosarcoma cells. A cytopathic effect was observed in a monitor linked with an inverted microscope after an incubation period of 4 to 5 days. The neutralization titers were read as the highest dilution that could result in a 50% reduction in the cytopathic effect. Each test sample was run simultaneously with cell control, serum control, and virus back titration. The starting dilution was 1:8; the cutoff level of seropositivity was set at 8. Seven EV71 strains were used for neutralization assay, including 4 Taiwan strains (C4a/70516/TW/08, B4/E59P2/TW/02, B5/141/TW/08, C5/575/TW/07) and 3 Vietnam strains (C4a/HCM82/VN/11, C4a/HCM120/VN/11, C5/HCM84/VN/11).

**Table 1:** Enterovirus 71 patients providing post-infection sera used for neutralization Assay.

**Patient ID**	**Onset date**	**Gender**	**Age (yr)**	**Symptom**	**Virus Genotype**	**Country**
C4a/EV95493/TW/10	1-Jun-10	M	2.73	HFMD	C4a	Taiwan
B4/CG814/01	5-Dec-01	F	1.58	HFMD	B4	Taiwan
B5/141/TW/08	7-Jul-08	F	1.62	Herpangina	B5	Taiwan

## Results

**Figure 1:**
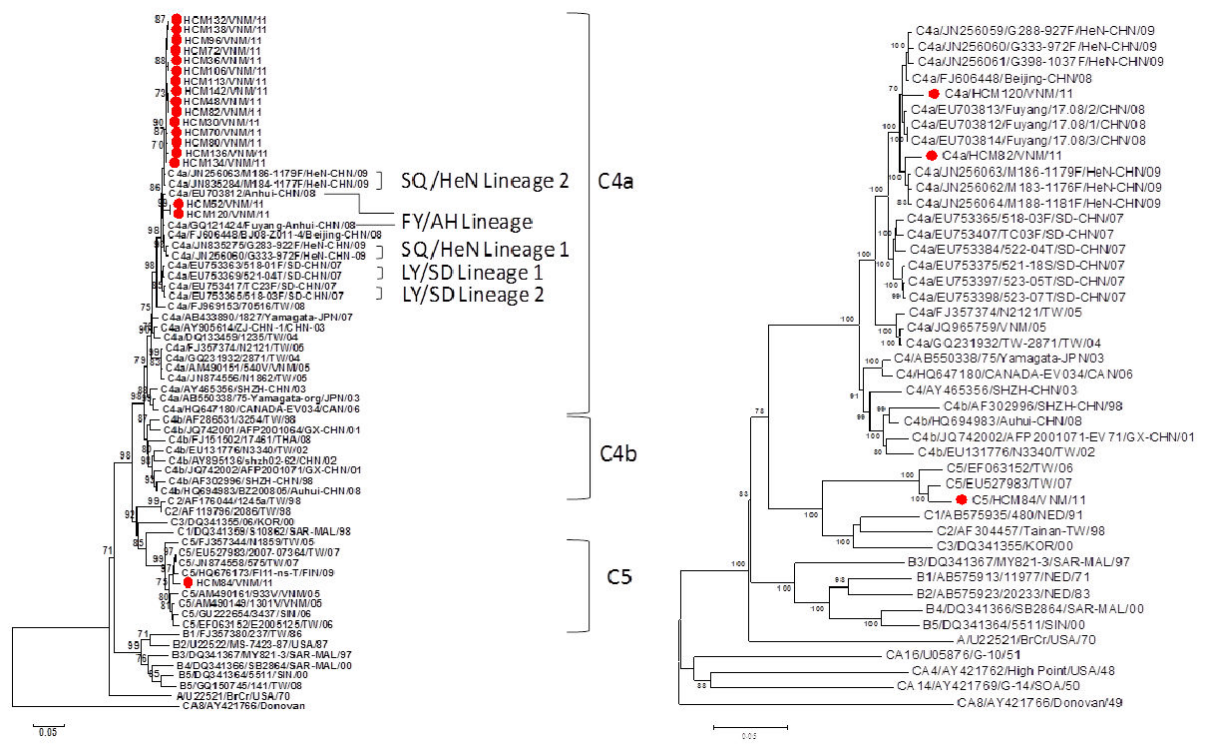
Phylogenetic relationships of EV71 viruses isolated in this study and reference strains using VP1 sequences (A) and full genomes (B). Backgrounds of the EV71 viruses genotyped in this study and the reference viruses were provided in Table 1 and Table S1 [15], respectively. The prototype coxsackievirus A16 (CA16G10) was used as the outgroup virus. The phylogenetic tree was constructed using the neighbor-joining method. Bootstrap values (>70%) are shown as percentage derived from 1,000 sampling at the nodes of the tree. Scale bar denotes number of nucleotide substitutions per site along the branches. Red dot indicates the viruses isolated and sequenced in this study.

In 2011, 148 HFMD inpatients were tested and 65 of them (43%) were positive for enterovirus by immunofluorescience assay, including 36 EV71 patients. Complete VP1 genes (891 nucleotides) of 18 EV71 isolates were sequenced for phylogenetic analysis. Based on phylogenetic analysis of complete VP1 genes, 94% (17/18) and 6% (1/18) of EV71 isolates are genotype C4a and C5, respectively (Table 2). Overall, the C4a viruses are phylogenetically related to the C4a viruses circulating in China and the only C5 virus is related to the C5 viruses isolated in Vietnam in 2005 and Taiwan in 2007 (Figure 1). Interestingly, 17 C4a virus strains isolated in Ho Chi Minh City could be classified into 2 groups including 15 C4a viruses in group one and 2 C4a viruses (virus ID HCM52 and HCM120) in group two. Genetic differences between and within VP1 genes of these two C4a groups were 2.6~2.9% and 0.2~0.3%, respectively. Therefore, full genome analysis (~7000 nucleotides) of three EV71 isolates including one virus from C4a group one (virus ID HCM82), one virus from C4a group two (virus ID HCM120) and the only C5 virus (virus ID HCM84), were further conducted to understand molecular evolution of the 2011 EV71 isolates in southern Vietnam. Based on BLAST analysis of full genome, the HCM82 virus is most similar to a C4a virus isolated in Henan China in 2009 (accession no. JN252063, nucleotide identity 99%), the HCM120 virus is most similar to a C4a virus isolated in Beijing, China in 2008 (accession no. FJ606448, nucleotide identity 98%), and the HCM84 virus is most similar to a C5 virus isolated in Taiwan in 2007 (accession no. EU527983, nucleotide identity 98%). Phylogenetic analysis of full genome revealed similar findings to that of the BLAST analysis (Figure 1).

**Table 2:** Background of the EV71 viruses isolated and genotyped in Ho Chi Minh City, Vietnam, 2011.

Virus ID	Accession no.	Genotype	Age	Clinical stages
HCM30	KC222957	C4a	1 yr	I
HCM36	KC222958	C4a	2 yr	IIA
HCM48	KC222959	C4a	2 yr	IIB
HCM 52	KC222960	C4a	2 yr	IIB
HCM 70	KC222961	C4a	2 yr	IIA
HCM 72	KC222962	C4a	3 yr	IIB
HCM 80	KC222963	C4a	3 yr	IIB
HCM 82	KC222964	C4a	3 yr	IIB
HCM 84	KC222965	C5	1 yr	IIA
HCM 96	KC222966	C4a	3 yr	IIB
HCM 106	KC222967	C4a	3 yr	IV
HCM 113	KC222968	C4a	2 yr	IV
HCM 120	KC222969	C4a	2 yr	IIB
HCM 132	KC222970	C4a	3 yr	I
HCM 134	KC222971	C4a	3 yr	III
HCM 136	KC222972	C4a	4 yr	III
HCM 138	KC222973	C4a	8 Mo	III
HCM 142	KC222974	C4a	1 yr	III

EV71 genotype C4 viruses could be classified into C4a and C4b. The EV71 genotype C4b viruses were first detected in China and Taiwan in 1998 with unknown origins and were later identified as recombinants among multiple enteroviruses in human enterovirus species A [9,15]. The C4b viruses continued to spread sporadically in Asia afterward and evolved into C4a genotype in 2003 in China. In 2004, the C4a viruses caused a large-scale epidemic in Taiwan but disappeared in Taiwan in 2006 [3]. Since 2007, the C4a viruses have become the predominant genotype with 5 different lineages and have repeatedly caused several large-scale epidemics in China [15]. Based on phylogenetic analysis of VP1 genes and full genomes, the C4a viruses detected in HCM in 2011 belong to two lineages which could have been separately introduced from China.

**Figure 2:**
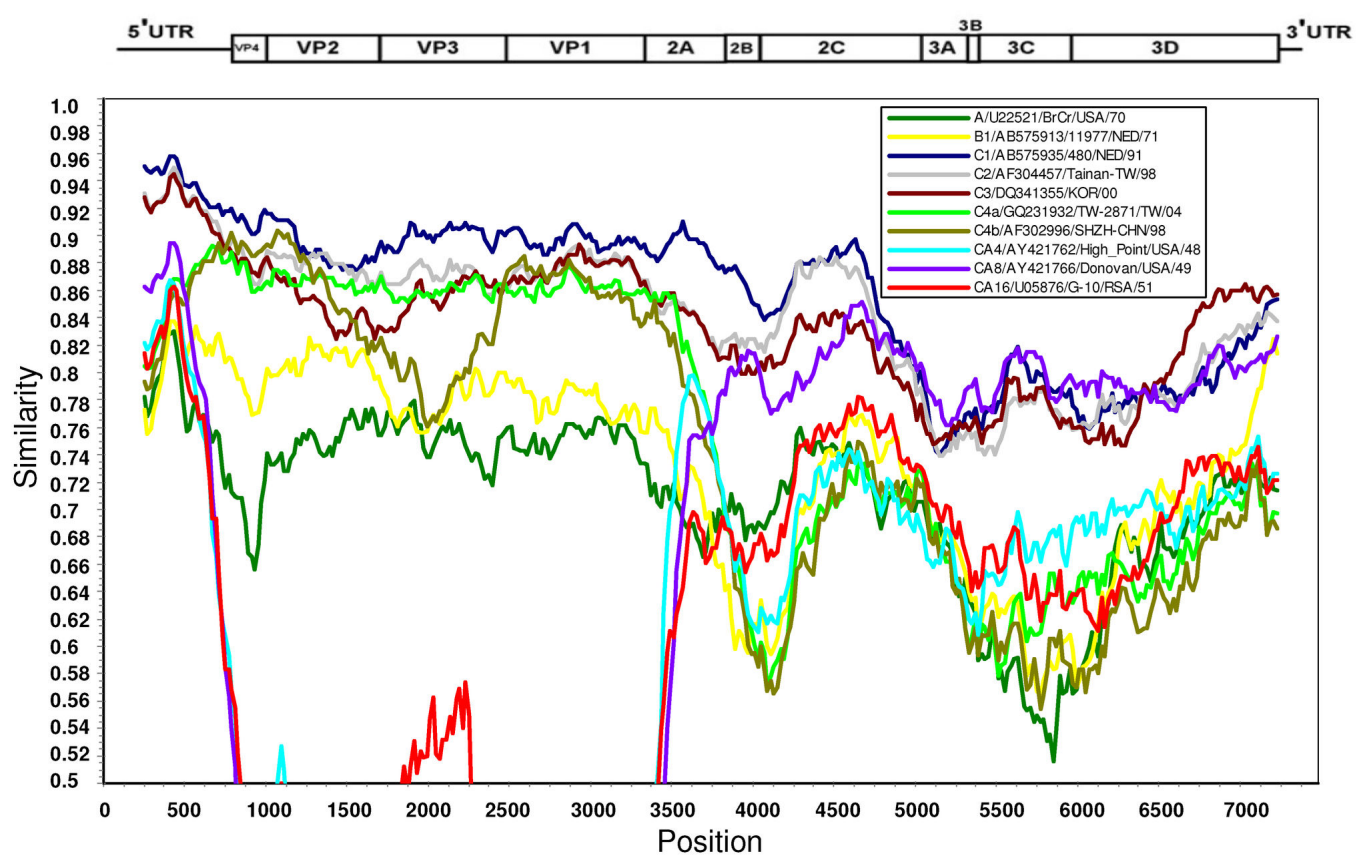
SimPlot analysis of EV71 C5 virus isolated in HCM City, Vietnam, 2011. The query virus is HCM84-VNM-2011 and four potential parental (EV71 genotype C1, C2 and C3, and CA8) viruses identified through BLAST and phylogenetic analysis and six reference viruses were selected for the SimPlot analysis.

So far, only two C5 viruses isolated in Taiwan in 2006 and 2007 have full genome sequence available in public domain, which were highly identical to the C5 virus isolated in Vietnam in 2011. The evolution pathway of C5 viruses has not been well characterized ant it is very likely through gene recombination. To further detect occurrence of genetic recombination, full genome sequence of the Vietnam C5 isolate was divided into P1 (nucleotide 744~3329), P2 (nucleotide 3330~5063), and P3 segments (nucleotide 5064~7322) for BLAST and phylogenetic analysis. These analyses indicated that P1 and P2 genes of the C5 viruses are closed to that of EV71 genotypes C1, C2 and C3 viruses but source of P3 gene could not be identified (Table 3). SimPlot analysis of full genome further confirmed the possibility of gene recombination at P3 gene (Figure 2). P3 gene of the C5 viruses could be derived from other human enterovirus specie A viruses such as Coxsakievirus A8 but it needs more full genome sequence data of early isolates to confirm.

**Table 3:** Nucleotide identity (%) between C5/HCM84/VN/11 and reference enterovirus strains.

Virus ID	5'UTR	VP4	VP2	VP3	VP1	2A	2B	2C	3A	3B	3C	3D	3'UTR
A/U22521/BrCr/USA/70	79.9	80.7	80.7	80.4	80.5	77.8	77.8	79.5	75.6	71.2	72.1	77.8	77.3
B0/AB575912/10857/NED/66	82.8	83.6	83.7	82.5	82.7	80.4	74.4	79.2	77.1	78.8	76.3	80.8	85
B1/AB575913/11977/NED/71	82.6	83.6	84	82	82.2	79.6	73.4	79.9	73.6	78.8	73.2	79.9	77.3
B1/FJ357380_237-TW86	81.3	83.1	84.1	81.3	82	79.8	74.7	78.7	72.9	78.8	74.7	78.2	72.7
B2/AB575923/20233/NED/83	83	81.2	83.6	81.5	81.6	80.7	75.4	79.8	72.1	75.8	74	80.7	61.5
B2/U22522/MS-7423-87/USA/87	82.3	82.6	83.2	82.1	81.8	80.4	75.8	79	72.5	75.8	73.6	80.3	77.3
B3/DQ341367/MY821-3/SAR-MAL/97	84.2	85	82.7	81.4	81.9	78.9	74.4	79.3	73.6	81.8	71.8	77.3	68.2
B3/DQ341354/3799/SIN/98	84.1	85	82.5	81.8	82.2	78.9	74.1	78.8	73.6	80.3	72.3	77.3	63.6
B4/DQ341366/SB2864/SAR-MAL/00	82.7	82.1	82.8	82.2	82.3	78.2	74.1	78.9	74	77.3	73.6	80.9	81.8
B4/AF316321/5865/sin/000009/SIN/00	82.3	84.1	81.8	82	82.8	79.3	73.7	78.8	74	77.3	73.2	80.7	81.8
B5/DQ341364/5511/SIN/00	82.9	85	82.2	82.2	82.4	78.2	73.4	79.3	75.2	75.8	73.6	81.2	77.3
B5/EU527985/2007-08747/TW/07	84.1	82.1	81.6	81.5	83.2	77.3	73.7	78.9	75.6	77.3	74.5	80.7	72.7
C1/AB575935/480/NED/91	93.3	93.2	90	90.9	90.3	91.1	86.9	87	77.5	87.9	83.2	83.5	95.2
C1/HQ647172/EV063_04/CAN/94	91.7	94.2	89.5	90.6	89.1	89.8	87.9	85.3	79.8	86.4	83.2	83.2	90.9
C2/AF304457/Tainan/5746/98	90.2	91.3	88.6	88.7	88.9	86.7	83.5	86.5	78.3	80.3	82.5	83.4	90.9
C2/DQ341357/7F-AUS-6-99	90.6	92.8	87.9	88.4	89.2	86.7	84.5	85.9	77.9	81.8	81.8	83.5	90.9
C3/DQ341355/06/KOR/00	89.8	90.8	86.4	87.6	89	86.4	83.2	84.4	79.5	80.3	82.9	84.3	90.9
C4b/AF302996/SHZH-CHN/98	84.7	88.4	88.6	83.6	87.9	83.1	73.4	78.1	74.8	77.3	72.5	77	54.5
C4a/GQ231932/TW/2871/TW/04	85.7	86	88.3	88.2	87.5	84.9	73.7	78.1	73.6	77.3	74.9	77.7	55
C4a/GQ231929/TW/2728/04	85.9	87	87.9	87.7	87.3	84.9	74.1	78.1	74	77.3	75.4	77.6	55
C5/EU527983/2007-07364/TW/07	97.7	98.6	98.7	97.9	98.4	98	97.3	97	98.1	98.5	96.7	97.6	95.5
CVA8/AY421766/Donovan/USA/49	85.2	63.8	64	66.5	59.2	81.6	82.8	84.1	80.6	75.8	84.2	83.3	95.5
CA16/U05876/G-10	81.6	67.1	69.6	71.9	63.1	78.2	76.1	81	76	78.8	75.6	78.7	55

To characterize antigenic variations among genotypes C4a, and C5 viruses isolated in Vietnam and Taiwan, sera collected from Taiwanese children infected with C4a, B4 and B5 were employed to measure cross-reactive neutralizing antibody titers against these EV71 viruses. As shown in Table 4, the C4a and C5 viruses isolated in Vietnam and Taiwan have similar antigenic profiles and could be neutralized by the post-infection children sera.

**Table 4:** Cross-reactive neutralizing antibody titers in post-infection children sera against EV71 viruses isolated in Taiwan and Vietnam.

	Neutralizing antibody titers in post-infection children sera		
Virus strain	C4a/EV95493/TW/10	B4/CG814/01	B5/141/TW/08
C4a/70516/TW/08	**1024**	32	128
B4/E59P2/TW/02	1024	**512**	256
B5/141/TW/08	1024	512	**256**
C5/575/TW/07	1024	256	256
C4a/HCM82/VN/11	512	128	128
C4a/HCM120/VN/11	256	16	128
C5/HCM84/VN/11	512	256	64

## Discussion

Since 1997, several EV71 genotypes have caused large-scale epidemics with fatal cases in Asian countries [3]. The EV71 genotype C5 viruses were first identified in Vietnam in 2003 and later detected in Taiwan in 2005, in Singapore in 2006 and in Finland in 2009 but only caused large-scale epidemics with fatal cases in Vietnam in 2005 [5,16,17]. The reason for this phenomenon is not clear and needs international collaborations to clarify. In addition, origin and evolution of EV71 genotype C5 viruses have not been well understood and comprehensive genome studies are desirable.

In a clinical investigation of HFMD epidemic in Southern Vietnam in 2011, partial VP1 sequences of 15 EV71 isolates were analyzed and 13 of them (87%) were C4a genotype and the remain 2 strains were C5 genotype, which are similar to the finding in our study [7]. However, complete genome sequencing and antigenic analysis were not conducted in the study. In addition, this clinical investigation did not conduct antigenic analysis which has been performed in our study. Although, large-scale EV71 epidemics in southern Vietnam were only reported in 2005 and 2011, EV71 were active in young children in HCM City during 2006~2009 based on serological and hospital-based enterovirus surveillance studies [18,19]. Moreover, it is well documented that molecular methods are much more sensitive than virus isolation for detecting enterovirus infections [20,21]. Therefore, enterovirus surveillance in endemic countries should combine virus culture, molecular methods and serology to well characterize disease burden.

International spreading of EV71 is common and may play an important role for reemergence of large-scale epidemics in Asia [3]. For example, EV71 genotype B5 viruses spread from Southeastern Asia to Taiwan in 2007 and caused a nation-wide epidemic in 2008 [9,13]. In the current study, EV71 genotype C4a viruses spread from China to Vietnam and caused a large-scale epidemic in HCM City and southern Vietnam in 2011. Recently, Western Pacific Regional Office of the World Health Organization has established a HFMD reporting system to collect monthly HFMD cases no. from China, Hong Kong, Macao, Japan, Republic of Korea, Singapore and Vietnam (www.wpro.who.int). However, the reporting systems vary country by country and do not include virus identification data, which make international comparisons impossible. As learnt from global influenza surveillance system, an international enterovirus surveillance system with harmonized clinical definitions and virus identification data is urgently desirable to understand epidemiology and disease burden of EV71 in Asia.

Based on serological analysis using hyperimmune animal antisera, EV71 has only one serotype. Recently, antigenic variations between different genotypes could be detected using post-infection human sera but did not have a clear pattern [9,13]. In this study, genotype C4a and B5 viruses isolated in Vietnam and Taiwan have similar antigenic profiles. Currently, different vaccine strains are being evaluated in China (genotype C4a), Singapore (genotype B2) and Taiwan (genotype B4) [3]. It would be important to monitor cross-reactive neutralizing antibody profiles using sera collected from vaccinated children in the future. Moreover, longitudinal enterovirus surveillance data are critical to formulate vaccination policy [9,14] and are warranted in Vietnam.

## Supporting Information

Table S1 List of reference virus strains used to conduct phylogenetic analysis of full genome and VP1 sequences. (DOC)

Table S2 Primers used for EV71VP1 and complete genome amplification and sequencing*. (DOC)
