# Supplementary material for: Genetic and Antigenic Characterization of Enterovirus 71 in Ho Chi Minh City, Vietnam, 2011
Source: PLoS One. 2013 Jul 29;8(7):e69895. doi: 10.1371/journal.pone.0069895 (PMC3726754; doi:10.1371/journal.pone.0069895)
Supplement: Table S1 — (DOC) [file pone.0069895.s001.doc]

Supplemental Table S1. List of reference virus strains used to conduct phylogenetic analysis of full genome and VP1 sequences.

| Virus ID | Accession no. | Genotype | Gene | Year | Country |
| --- | --- | --- | --- | --- | --- |
| BrCr-CA-70 | U22521 | A | Full genome | 1970 | USA |
| 11977 | AB575913 | B1 | Full genome | 1971 | Netherland |
| 237 | FJ357380 | B1 | VP1 | 1986 | Taiwan |
| 20233 | AB575923 | B2 | Full genome | 1983 | Netherland |
| MS-7423-87 | U22522 | B2 | VP1 | 1987 | USA |
| MY821-3 | DQ341367 | B3 | Full genome | 1997 | Malaysia |
| SB2864-SAR-00 | DQ341366 | B4 | Full genome | 2000 | Malaysia |
| 5511 | DQ341364 | B5 | Full genome | 2000 | Singapore |
| 141 | GQ150745 | B5 | VP1 | 2008 | Taiwan |
| 480 | AB575935 | C1 | Full genome | 1991 | Netherland |
| S10862 | DQ341359 | C1 | VP1 | 1998 | Malaysia |
| Tainan/5746/98 | AF304457 | C2 | Full genome | 1998 | Taiwan |
| 1245a | AF176044 | C2 | VP1 | 1998 | Taiwan |
| 2086 | AF119796 | C2 | VP1 | 1998 | Taiwan |
| 6 | DQ341355 | C3 | Full genome | 2000 | Korea |
| ZJ-CHN-1 | AY905614 | C4a | VP1 | 2003 | China |
| 1235 | DQ133459 | C4a | VP1 | 2004 | Taiwan |
| TW/2871 | GQ231932 | C4a | Full genome | 2004 | Taiwan |
| N2121-TW05 | FJ357374 | C4a | Full genome | 2005 | Taiwan |
| N1862 | JN874556 | C4a | VP1 | 2005 | Taiwan |
| 540V | JQ965759 | C4a | Full genome | 2005 | Vietnam |
| 518-01F | EU753363 | C4a | VP1 | 2007 | China |
| 518-03F | EU753365 | C4a | Full genome | 2007 | China |
| 521-18S | EU753375 | C4a | Full genome | 2007 | China |
| 521-04T | EU753369 | C4a | VP1 | 2007 | China |
| 522-04T | EU753384 | C4a | Full genome | 2007 | China |
| 523-05T | EU753397 | C4a | Full genome | 2007 | China |
| 523-07T | EU753398 | C4a | Full genome | 2007 | China |
| TC03F | EU753407 | C4a | Full genome | 2007 | China |
| TC23F | EU753417 | C4a | VP1 | 2007 | China |
| 1827 | AB433890 | C4a | VP1 | 2007 | Japan |
| Fuyang.Anhui/17.08/1 | EU703812 | C4a | Full genome | 2008 | China |
| Fuyang.Anhui/17.08/2 | EU703813 | C4a | Full genome | 2008 | China |
| Fuyang.Anhui/17.08/3 | EU703814 | C4a | Full genome | 2008 | China |
| Fuyang.Anhui/19.08/7 | GQ121424 | C4a | VP1 | 2008 | China |
| 70516 | FJ969153 | C4a | VP1 | 2008 | Taiwan |
| BJ08-Z011-4 | FJ606448 | C4a | Full genome | 2009 | China |
| G183-922F | JN835275 | C4a | VP1 | 2009 | China |
| G288-927F | JN256059 | C4a | Full genome | 2009 | China |
| G333-972F | JN256060 | C4a | Full genome | 2009 | China |
| G398-1037F | JN256061 | C4a | Full genome | 2009 | China |
| M183-1176F | JN256062 | C4a | Full genome | 2009 | China |
| M186-1179F | JN256063 | C4a | Full genome | 2009 | China |
| M186-1177F | JN835284 | C4a | VP1 | 2009 | China |
| M188-1181F | JN256064 | C4a | Full genome | 2009 | China |
| 75-Yamagata-org | AB550338 | C4* | Full genome | 2003 | Japan |
| SHZH-CHN | AY465356 | C4* | Full genome | 2003 | China |
| CANADA-EV034 | HQ647180 | C4* | Full genome | 2006 | Canada |
| SHZH98 | AF302996 | C4b | Full genome | 1998 | China |
| 3254 | AF286531 | C4b | VP1 | 1998 | Taiwan |
| AFP2001064 | JQ742001 | C4b | VP1 | 2001 | China |
| AFP2001071 | JQ742002 | C4b | Full genome | 2001 | China |
| shzh02-62 | AY895136 | C4b | VP1 | 2002 | China |
| N3340 | EU131776 | C4b | Full genome | 2002 | Taiwan |
| SHZH-CHN | AY465356 | C4b | Full genome | 2003 | China |
| 75 | AB550338 | C4b | Full genome | 2003 | Japan |
| CANADA-EV034 | HQ647180 | C4b | Full genome | 2006 | Canada |
| BZ200805 | HQ694983 | C4b | Full genome | 2008 | China |
| 17461 | FJ151502 | C4b | VP1 | 2008 | Thailand |
| N1859 | FJ357344 | C5 | VP1 | 2005 | Taiwan |
| 933V | AM490161 | C5 | VP1 | 2005 | Vietnam |
| 1301V | AM490149 | C5 | VP1 | 2005 | Vietnam |
| 3437 | GU222654 | C5 | VP1 | 2006 | Singapore |
| E2005125 | EF063152 | C5 | Full genome | 2006 | Taiwan |
| 2007-07364 | EU527983 | C5 | Full genome | 2007 | Taiwan |
| 575 | JN874558 | C5 | VP1 | 2007 | Taiwan |
| FI11-ns-T | HQ676173 | C5 | VP1 | 2009 | Finland |
| High Point | AY421762 | CVA-4 | Full genome | 1948 | USA |
| Donovan | AY421766 | CVA-8 | Full genome | 1949 | USA |
| G-14 | AY421769 | CVA-14 | Full genome | 1950 | RSA |
| G-10 | U05876 | CVA-16 | Full genome | 1951 | RSA |

*The virus was grouped into C4a lineage based on its VP1 sequence but was grouped into C4b based on its complete genome with phylogenetic analysis.

RSA, Republic of South Africa
