# Supplementary material for: Genetic and Antigenic Characterization of Enterovirus 71 in Ho Chi Minh City, Vietnam, 2011
Source: PLoS One. 2013 Jul 29;8(7):e69895. doi: 10.1371/journal.pone.0069895 (PMC3726754; doi:10.1371/journal.pone.0069895)
Supplement: Table S2 — (DOC) [file pone.0069895.s002.doc]

Supplemental Table S2. Primers used for EV71VP1 and complete genome amplification and sequencing.

| Primer | Position^a^ | Sequence (5'-3') ^b^ | Target region | Polarity |
| --- | --- | --- | --- | --- |
| EV71VP1 amplification and sequencing | | | | |
| EV71-F1-VP3^cd^ | 2217-2236 | ATWCCATGGATCAGYAAYAC | VP3 | sense |
| EV71-R1-2A^cd^ | 3553-3534 | CTGACYGGRTAGTGYTTTCT | 2A | antisense |
| EV71 complete genome amplification and sequencing | | | | |
| EV-5UTR-34F^cde^ | 34-52 | GGCCCACTGGGCGCTAGCA | 5’-UTR | sense |
| EV-5UTR-541F^cd^ | 544-568 | GACTACTTTGGGTGTCCGTGTTTCC | 5’-UTR | sense |
| EV-5UTR-503R^cd^ | 505-529 | GTTACCCGTTACGACACACTACCCG | 5’-UTR | antisense |
| EV-5UTR-355F^cd^ | 375-396 | CCTGCCCATGGAGAAATCCATG | 5’-UTR | sense |
| EV-5UTR-854R^cd^ | 851-872 | TTTGCCTGCTGTGGCGGCATAG | 5’-UTR | antisense |
| EV71-633F^cd^ | 633-653 | TGGYCATCCRGTGTSHAYCAG | 5’-UTR | sense |
| EV71-3540R^cd^ | 3553-3534 | CTGACYGGRTAGTGYTTTCT | 2A | antisense |
| EV71S-1443F^d^ | 1443-1462 | GTGCTCGAYGCTGGBATYCC | VP2 | sense |
| EV71-3024F^cd^ | 3025-3044 | CACCTGCGAGTGCTTATCAA | VP1 | sense |
| EV71-5838R^cd^ | 5839-5820 | ACCACTCCTCCACACTGTCC | 3C | antisense |
| EV71-3743F^d^ | 3722-3739 | TGAGGGATCTYYTGTGGT | 2A | sense |
| EV71S-4428F^d^ | 4428-4447 | CACCGWATTGAACCTGTRTG | 2C | sense |
| EV9-5131F^cd^ | 5128-5151 | GCGATCTCCTCGCTAGTGTAGATA | 3A | sense |
| EV71-7410R^cd^ | 7391-7408 | GCTATTCTGGTTATAACA | 3’-UTR | antisense |
| EV71S-5790F^d^ | 5790-5806 | ACYATGATGTAYAAYTT | 3C | sense |
| EV71S-6297F^d^ | 6297-6316 | AGYGCHYTDGGYATMAAGAA | 3C | sense |
| Oligo-T^c^ | 7049~ | This primer contains 12-18 dT bases. | Poly-A tail | antisense |

^a^ The positions of primers are those relative to the genome of EV71 BrCr (GenBank accession number U22521).

^b^ Degenerate bases followed nucleotide IUB codes.

^c^ RT-PCR primer

^d^ Sequencing primer

^e^ Published by Wang et al in J Clin Microbiol, 2002
